# Hepatocellular carcinoma

**DOI:** 10.1054/bjoc.2001.1769

**Published:** 2001-05

**Authors:** M Giovannini, D Elias, G Monges, J L Raoul, P Rougier

**Affiliations:** Institut Paoli-Calmettes, Marseille; Institut Gustave Roussy, Villejuif; Centre Eugène Marquis, Rennes; Hôpital Anbroise Paré, Boulogne, France


					The incidence of hepatocellular carcinoma (HCC) has been rising
steadily and varies between 2 and 5 per 100 000 per year in Europe
and the USA. These tumours are associated with cirrhosis in 80%
of cases.
The major goals in this disease are to clarify the natural history
of HCC, to evaluate the efficacy of a wide range of treatments and
to carry out controlled trials in order to define which treatments are
best in terms of risk and benefit.
These recommendations relate to the management of primary
hepatocellular carcinoma and its variants (e.g. fibrolamellar, clear
cell and giant cell carcinoma) in both cirrhotic and normal livers.
These guidelines were validated in June 1999 by the working
group and an update is planned for 2001.
DIAGNOSIS
With the exception of the cirrhotic patient with an alpha-
fetoprotein (AFP) level greater than 500 mg ml-1
, the diagnosis of
hepatocellular carcinoma (HCC) is based on the histopathological
examination of one or more liver sample. These are obtained at
open surgery, by laparoscopy or by ultrasound or CT scan-guided
biopsy (standard). Fine-needle aspiration for cytology is an option
for the diagnosis of HCC and can be considered if a liver biopsy is
not possible. The ease of diagnosis depends on the quality and
nature of the biopsy specimens and the histologic type of the
lesions. If the level of AFP is normal in a cirrhotic patient, the
level of gamma-carboxyprothrombin (in patients without a
Vitamin K deficiency) can be measured (option).
STAGING
Assessment of locoregional extension depends on clinical exami-
nation, liver ultrasonography and hepatic CT scanning (standard).
MRI of the liver is an option. Standard investigations to assess
distant spread include a chest X-ray (CXR), a spiral CT and a bone
scan if this is clinically indicated. If a liver transplant is planned
and the CXR is normal, a thoracic CT scan should be done. The
evaluation of hepatic function depends on liver enzyme levels,
serum albumin and a coagulation screen (standard). Endoscopy
must be undertaken in the case of hepatic cirrhosis to exclude the
presence of oesophagogastric varices (standard). In the case of
diagnostic difficulty in differentiating an adenoma, a cholangio-
carcinoma or a metastatic lesion, immunohistochemical staining to
identify keratin subtypes and/or reticulin staining can be consid-
ered.
CLASSIFICATION
Primary HCC is classified according to the WHO classification
(standard). There is no standard classification for the differentiation
or grade of HCC. This can be done according to the grading of
Edmonson or following the recommendations of the WHO
(option). The standard classification for patients having a surgical
resection is the TNM classification, where multiple tumours,
large-diameter tumours and the invasion of vasculature and nodes
are adverse prognostic factors. The associated pathological stan-
dard is the classification of Child-Pugh. For non-operable
patients, the clinical classification system of Okuda can be used.
This is useful but not without problems.
TREATMENT MODALITIES
Resection is indicated for HCC in a cirrhotic liver when the
tumour is single and non-metastatic and if there is no portal inva-
sion (level of evidence C). The liver function must allow for the
type of excision necessary according to the localization and size of
the tumour. The majority of teams will undertake liver transplanta-
tion for unifocal HCC of less than 3 cm in diameters that is
secondary to cirrhosis and has been discovered by chance.
In the case of HCC in a healthy liver, excision by partial hepat-
ectomy is the only surgical modality to be considered. It should be
undertaken where there is no vascular involvement, no extra-
hepatic spread and when the tumour is uni-focal. Elective incom-
plete excision should be avoided. Percutaneous techniques
(intratumoral injections of absolute alcohol or acetic acid) or ultra-
sonic thermo-ablation are alternatives when surgery is not possible
(level of evidence C). Randomized studies are necessary to deter-
mine the exact place of these techniques in the treatment of HCC
as compared to surgery and other techniques of local treatment.
Chemo-embolization is not recommended for the treatment of
HCC (option, level of evidence B). This therapy has no proven
benefit with respect to survival over no therapy or systemic
chemotherapy. This technique must be evaluated within random-
ized trials of adjuvant or neoadjuvant treatment.
`In situ' radiotherapy is an option for the treatment of inoper-
able patients with portal thrombosis (level of evidence C).
Chemotherapy represents an area of research for the treatment
of inoperable HCC, but the results to date are inferior to those of
other treatment modalities.
Immunotherapy is an option for the treatment of inoperable
hepatocellular carcinoma (level of evidence C). It has questionable
efficacy when compared to systemic chemotherapy. It place as
adjuvant treatment within complex therapeutic strategies must be
evaluated in randomized trials.
Hormone therapy represents an option for palliative treatment in
patients with inoperable or metastatic hepatocellular carcinoma in
whom neither percutaneous techniques, chemo-embolization, nor
in situ radiotherapy are possible (level of evidence C). The place
of somatostatin must be confirmed by prospective randomized
trials.
Hepatocellular carcinoma
M Giovannini1
, D Elias2
, G Monges1
, JL Raoul3
and P Rougier4
1
Institut Paoli-Calmettes, Marseille; 2
Institut Gustave Roussy, Villejuif; 3
Centre Eugene Marquis, Rennes; 4
Hopital Anbroise Pare, Boulogne, France
74
British Journal of Cancer (2001) 84 (Supplement 2), 74-77
(c) 2001 FNCLCC
doi: 10.1054/ bjoc.2001.1769, available online at http://www.idealibrary.com on
THERAPEUTIC STRATEGY
HCC in a cirrhotic liver
The objective of treatment in these patients is to improve survival
and/or to provide palliation. There are no standard therapeutic
approaches.
Unifocal HCC (Single lesion HCC <5 cm maximum
diameter)
Child-Pugh A disease Surgical excision, hepatic transplan-
tation and percutaneous techniques can be considered (options)
(Figure 1). It is recommended that surgical excision be undertaken
if at all possible and within a specialist setting (level of evidence
C). The role of adjuvant treatment remains to be established.
Child-Pugh B disease Hepatic transplantation, percutaneous
techniques, the use of radioactive lipioidal or chemo-embolization
can be considered (option) (Figure 1). It is recommended that the
place of hepatic transplantation be evaluated within a formal
protocol. Any surgery must be undertaken within a specialist
setting (level of evidence C). In the case of a Child-Pugh B lesion
of small size, percutaneous techniques are recommended (level of
evidence C).
Child-Pugh C disease Hepatic transplantation, hormone
therapy or best supportive care can be considered (option).
Unifocal HCC (Single lesion HCC > 5 cm maximum
diameter)
Child-Pugh A and B disease Liver resection, chemo-
embolization ( alcohol injection) and radioactive lipioidal can be
considered. If at all possible, resection should be undertaken
within a specialist centre (level of evidence C).
Child-Pugh C disease The objective of treatment is palliation.
There is no standard therapy. Hormone therapy or best supportive
care can be considered (option).
Multifocal HCC without portal thrombosis
Child-Pugh A and B If there are less or equal to three lesions
of less than 5 cm in diameter, surgical resection, transplantation
and percutaneous procedures can be considered (Figure 2). In
other cases, chemo-embolization or radioactive lipioidal injections
can be considered (option). Surgical excision is recommended for
peripheral tumours (level of evidence C), hepatic transplantation
for central tumours and percutaneous techniques for microtumours
(tumours of less than 5 cm diameter) (level of evidence C).
Child-Pugh C disease The objective of treatment in the
management of these patients is palliation. Hormone therapy or
symptomatic management alone can be considered (option)
(Figure 2).
Multifocal HCC with portal thrombosis
Child-Pugh A and B disease These patients have inoperable
disease. Radioactive lipioidal, chemo-lipioidal (without emboliza-
tion) and external-beam irradiation (for lateral tumours) can be
considered (option).
Child-Pugh C disease The objective of treatment is palliation.
Hormone therapy or symptomatic care can be considered (option).
Metastatic HCC
Child-Pugh A and B disease The objective of treatment is
palliation. Chemotherapy, hormone therapy or symptomatic care
can be considered (option) (Figure 3). Inclusion in ongoing thera-
peutic trials is recommended.
Hepatocellular carcinoma 75
British Journal of Cancer (2001) 84(Supplement 2), 74-77
(c) 2001 FNCLCC
SINGLE LESION NON-METASTATIC HCC IN A
CIRRHOTIC LIVER
yes no
< 5 cm?
CHILD-PUGH
A
CHILD-PUGH
B
CHILD-PUGH
C
CHILD-PUGH
A and B
CHILD-PUGH
C
Standard
there is no standard
Options
* surgical resection
* liver transplant
* percutaneous
* techniques
Standard
there is no standard
Options
* liver transplant
* percutaneous
techniques
* radioactive lipioidol
* hormone therapy
* symptomatic
treatment
Standard
there is no standard
Options
* liver transplant
* percutaneous
techniques
* hormone therapy
* symptomatic
treatment
Standard
there is no standard
Options
* surgical reaction
* chemo-embolization
* radioactive lipioidol
Standard
there is no standard
Options
* hormone therapy
* symptomatic
treatment
Figure 1 Unifocal HCC in a cirrhotic liver
76 M Giovannini et al
British Journal of Cancer (2001) 84(Supplement 2), 74-77 (c) 2001 FNCLCC
Child-Pugh C disease The objective of treatment is palliation.
Hormone therapy or symptomatic care can be considered (option).
HCC in a non-cirrhotic liver
Single peripheral lesions
Surgical excision by partial hepatectomy is standard treatment
(Figure 4).
Single central lesions
Surgical excision by partial hepatectomy is standard treatment
(Figure 4).
Multifocal disease
There is no standard treatment. Percutaneous techniques, chemo-
embolization or radioactive lipoidal can be considered (option)
(Figure 4).
Metastatic HCC
There is no standard treatment (Figure 3). Chemotherapy, high-
dose interferon, hormone therapy, surgical excision (if feasible) or
symptomatic treatment alone can be considered (option)
FOLLOW-UP
There is no consensus regarding the pattern or modalities of follow-up
in HCC apart from clinical examination. Hepatic ultrasonography,
measurement of alpha-fetoprotein, abdominal CT scan, CXR and
MRI imaging are all options. Surveillance should be planned
according to the treatment given.
MULTIFOCAL NON-METASTATIC HCC IN A
CIRRHOTIC LIVER
yes no
no yes
Portal thrombosis?
Diameter
< 5 cm, N < 3?
CHILD-PUGH
A and B
CHILD-PUGH
C
CHILD-PUGH
A and B
CHILD-PUGH
C
Standard
there is no standard
Options
* radioactive lipioidol
* chemolipioidol without
embolization
* extemal-beam
radiotherapy (lateral
tumours)
Standard
there is no standard
Options
* alcoholization
* surgical resection
* liver transplant
* chemo-embolization
* radioactive lipioidol
Standard
there is no standard
Options
* chemo-embolization
* radioactive lipioidol
Standard
there is no standard
Options
* hormone therapy
* symptomatic
treatment
Standard
there is no standard
Options
* hormone therapy
* symptomatic
treatment
Figure 2 Treatment of multifocal HCC in a cirrhotic liver
METASTATIC HCC
yes no
Non-cirrhotic liver?
yes no
CHILD-PUGH
C
CHILD-PUGH A or B
Standard
there is no standard
Options
* chemotherapy
* high-dose interferon
* hormone therapy
* symptomatic treatment Standard
there is no standard
Options
* hormone therapy
* symptomatic treatment
Figure 3 Treatment of metastatic HCC
Hepatocellular carcinoma 77
British Journal of Cancer (2001) 84(Supplement 2), 74-77
(c) 2001 FNCLCC
INTERNAL REVIEWERS
Y Becouarn (Institut Bergonie, Bordeaux), L Blanc (Centre
Francois Baclesse, Caen), E Cabarrot (Centre Claudius Regaud,
Toulouse), JR Delpero (Institut Paoli Calmettes, Marseille),
M Ducreux (Institut Gustave Roussy, Paris), M Fonck (Institut
Bergonie Bordeaux), J Fraisse (Centre Georges-Francois Leclerc,
Dijon), JY Herry (Centre Eugene Marquis, Rennes), A Kunlin
(Centre Henri Becquerel, Rouen), P Lasser (Institut Gustave
Roussy, Villejuif), G Lorimier (Centre Paul Papin, Angers),
G Mac Grogan (Institut Bergonie, Bordeaux), JC Machiavello
(Centre Antoine Lacassagne, Nice), B Meunier (Centre Eugene
Marquis, Rennes), P Rauch (Centre Alexis Vautrin, Nancy),
M Rivoire (Centre regional Leon Berard, Lyon), A Roche (Institut
Gustave Roussy, Villejuif), S Theobald (Institut Jean Godinot,
Reims), P Troufleau (Centre Alexis Vautrin, Nancy) and M Ychou
(Centre Val d'Aurelle, Montpellier).
EXTERNAL REVIEWERS
JM Ardiet (Clinique Saint-Jean, Lyon), S Baesjou (La Rochelle),
C Balabaud (Hopital Saint-Andre, Bordeaux), N Barbet (Macon),
C Bazin (CHU, Vandoeuvre-Les-Nancy), L Bedenne (CHRU,
Dijon), J Belfort (Le Mans), PM Bret (The Montreal General
Hospital, Montreal - Canada), P Brun (Hopital Hautepierre,
Strasbourg), P Collin (Clinique Courlancy, Nancy), R Coquard
(Clinique Saint-Jean, Lyon), JP Dujols (Centre de Radiotherapie et
d'Oncologie Medicale, Pau), JP Dutin (Centre Saint-Michel, La
Rochelle), F Economides (Dunkerque), PL Etienne (Clinique
Armoricaine, Saint-Brieuc), MP Farcy-Jacquet (Clinique
Armoricaine, Saint-Brieuc), M Flesch (Centre de Radiotherapie du
Parc, Dijon), D Fric (Clinique du Mail, Grenoble), G Ganem
(Centre Jean Bernard, Le Mans), C Georgeac (Clinique Saint-
Come, Le Mans), M Gignoux (Hopital Cote de Nacre, Caen),
S Greget (Clinique Sainte-Clotilde, Sainte-Clotilde), F Guichard
(Bordeaux), D Kamioner (Hopital de Chartres, Chartres),
D Langlois (Clinique Saint-Michel, La Rochelle), P Lucas
(Polyclinique Les Bleuets, Reims), P Martin (Clinique Saint-Jean,
Lyon), M Moro (Nice), J Pitre (Hopital Cochin, Paris), M Pouyet
(Hopital la Croix Rousse, Lyon), J Saric (Hopital Saint-Andre,
Bordeaux), P Segol (CHU Cote de Nacre, Caen), R Soleilhac
(Clinique Sainte-Clotilde, Sainte-Clotilde), C Trepo (Hotel Dieu,
Lyon), JC Trinchet (Hopital Jean-Verdier, Bondy) and C Zaranis
(Clinique du Mail, La Rochelle).
NON-METASTATIC HCC IN A NON-CIRRHOTIC LIVER
yes no
Multifocal disease?
yes no
Central localization?
Standard
there is no standard
Options
* percutaneous technique
* chemo-embolization
* radioctive lipioidol
* surgery if possible
Standard
surgery: partial hepatectomy
Standard
surgery: partial hepatectomy
Figure 4 Multifocal HCC in a non-cirrhotic liver

				

